# Microwave-Enhanced Crystalline Properties of Zinc Ferrite Nanoparticles

**DOI:** 10.3390/nano12172987

**Published:** 2022-08-29

**Authors:** Martin Ochmann, Vlastimil Vrba, Josef Kopp, Tomáš Ingr, Ondřej Malina, Libor Machala

**Affiliations:** 1 Department of Experimental Physics, Faculty of Science, Palacký University Olomouc, 17. listopadu 1192/12, 779 00 Olomouc, Czech Republic; 2 Regional Centre of Advanced Technologies and Materials, Czech Advanced Technology and Research Institute (CATRIN), Palacký University Olomouc, Šlechtitelů 27, 779 00 Olomouc, Czech Republic

**Keywords:** microwave synthesis, zinc ferrite, inversion factor, coprecipitation method, crystal growth

## Abstract

Two series of ZnFe_2_O_4_ mixed cubic spinel nanoparticles were prepared by a coprecipitation method, where a solution of Fe^3+^ and Zn^2+^ was alkalised by a solution of NaOH. While the first series was prepared by a careful mixing of the two solutions, the microwave radiation was used to enhance the reaction in the other series of samples. The effect of the microwave heating on the properties of the prepared particles is investigated. X-ray powder diffraction (XRD), ^57^Fe Mössbauer spectroscopy and magnetometry were employed to prove the cubic structure and superparamagnetic behavior of the samples. The particle size in the range of nanometers was investigated by a transmission electron microscopy (TEM), and the N_2_ adsorption measurements were used to determine the BET area of the samples. The stoichiometry and the chemical purity were proven by energy dispersive spectroscopy (EDS). Additionally, the inversion factor was determined using the low temperature Mössbauer spectra in the external magnetic field. The microwave heating had a significant effect on the mean coherent length. On the other hand, it had a lesser influence on the size and BET surface area of the prepared nanoparticles.

## 1. Introduction

Cubic spinel ferrites represent a class of highly stable materials [1], which provide interesting electrical [2,3,4], magnetic [5,6], optical [7,8] and catalytic features [9,10,11]. The applicability of these materials can be further improved by tuning their properties via suitable preparation conditions [1,12]. The most investigated properties are the particle size, mean coherent length (MCL) [12], chemical composition, specific surface area or inversion factor [13,14]. For example, depending on their individual properties, spinel ferrites could be used in Fisher–Tropsch synthesis (a catalytic transformation towards carbohydrates) [15,16], where the chemical composition and surface area play a dominant role in the catalytic effectiveness. Additionally, the tuning of the inversion factor can enhance the catalytic performance even more [1,7]. Due to the hardness of iron oxides and chemical stability of ferrites, another possible application is surface coatings for the protection of steel [17,18].

Cubic ferrites can be generally considered as mixed cubic spinel materials, where the cation distribution exhibits two extreme cases: the normal spinel and the inverse spinel. The cation distribution of the zinc ferrite can be written as:

(1) $$\left({Zn}_{x}{Fe}_{1-x}\right)\left[{Zn}_{1-x}{Fe}_{1+x}\right]O_{4}$$

with the ratio of Zn^II^ and Fe^III^ being 1:2. The cations are distributed between the 8 tetrahedral and 16 octahedral sites, which are given (see Equation (1)) in round and square brackets, respectively [9]. The bulk zinc ferrite is a normal spinel with x=1, but as the particle size decreases, the inversion factor approaches the value of x=0, characterizing an inverse spinel. The inversion factor *x* could be determined from the distribution of iron cations between the tetrahedral and octahedral sites, leading to the following equation:

(2) $$\frac{N(Fe)}{N[Fe]}=\frac{1-x}{1+x}\;,$$

where the simplification of Equation (2) gives:

(3) $$x=\frac{N[Fe]-N(Fe)}{N[Fe]+N(Fe)}\;,$$

where N(Fe) represents the number of iron cations in tetrahedral sites and N[Fe] in octahedral sites.

Concerning the magnetic properties, most of the spinel ferrites can be classified as ferrimagnetic materials using Néel’s model of two sublattices. Nonetheless, the bulk zinc ferrite is paramagnetic at room temperature because, being the normal spinel, all Fe^III^ ions are located in octahedral sites only (Zn^II^ ions are diamagnetic). Thus, in bulk, room temperature ferromagnetic behaviour is observed only for substituted zinc ferrites [10]. However, zinc ferrite nanoparticles can exhibit magnetic behavior, which is caused by the cation distribution, where x≠1 [12]. Furthermore, the nanosized spinel ferrites can also exhibit superparamagnetism [6], which is characterized by its blocking temperature. Magnetic properties generally depend on a cationic substitution, the distribution of cations in sublattices or a particle size. It is also important to note that the observed magnetic behavior also depends on the specific time of measurement, where, in some cases, the ferrimagnetic behavior may be observed as paramagnetic- or ferromagnetic-like signals [12].

Mössbauer spectroscopy is a powerful tool that is able to determine the relative number of iron atoms in different local structural and electronic environments. The overall profile of Mössbauer spectra is influenced by the chemical state, local atomic ordering, or hyperfine magnetic field. Zinc ferrite, similarly to other nanosized spinel ferrites, usually results in a doublet in the room temperature spectrum. Although most ferrites exhibit sextets at larger particle sizes, zinc ferrite retains its doublet spectrum due to its bulk paramagnetic behavior. To resolve the magnetic ordering, Mössbauer spectroscopy needs to be combined with magnetometry. In most cases, it is necessary to conduct low-temperature Mössbauer spectroscopy measurements in the external magnetic field as well [13,14,19,20,21].

In the past, different synthesis procedures of spinel ferrites were reported [11,12]. One of the most used methods is a solid-state synthesis providing highly crystalline particles but with low specific surface areas [22]. These particles can be used, e.g., as a ceramic material for coils [23]. The combustion techniques result in spinel ferrites with similar properties because the final phase also comprises a high temperature treatment. Nonetheless, this synthesis route can provide nanoparticles having mean coherent lengths in the range of 30–70 nm, as shown by [24,25]. To prevent sintering and to fabricate highly crystalline nanoparticles, the usage of sol–gel synthesis is usually the best option [3,26]. However, this synthesis procedure can be time-, energy- or material-consuming. In addition, in some cases, environmentally hazardous materials need to be used as well. As sustainable and waste-free syntheses are preferred, the answer may lie in other wet-chemical techniques such as coprecipitation [27]. Coprecipitation results in nanocrystalline nanoparticles with relatively high specific surface areas [28], which are useful for various applications, including catalysis. As wet-chemical synthesis does not exceed the boiling temperature of a solvent, the resulting nanoparticles usually exhibit a low value of mean coherent length [29]. Another disadvantage is the possible presence of oxyhydroxides, hydroxides or adsorbed water in the prepared powder [1,7,30]. Enhancing the product properties that result from these wet-chemistry methods is a challenging process. A standard method involves removing the supernatant liquid and a subsequent heating of the prepared nanoparticles. However, influencing the synthesis process still in the solution is what usually limit these methods. One of the possible ways to enhance the reaction process is to use microwave radiation. Microwave heating can cause a very fast rise in the solution temperature, often resulting in a superheated solution. A higher mean coherent length can thus be expected in a relatively lower time frame compared to the mentioned wet-chemistry techniques. Moreover, the microwave-assisted syntheses do not cause sintering or extensive growth of the prepared nanoparticles, as suggested in [21,28]. These factors make the microwave-assisted coprecipitation method a green, very fast and unique synthesis route.

In this paper, we investigate the preparation of zinc ferrite nanoparticles on a series of microwave-assisted coprecipitation experiments. We combined the advantages of the classic wet synthesis technique (coprecipitation) with the effects of microwave radiation. To emphasize the significant benefits of the microwave-assisted synthesis, a series of experiments involving the classic coprecipitation synthesis at room temperature without the microwave field was conducted as well. The aim of this paper is to analyze the effect of the microwave irradiation time on the properties of the prepared zinc ferrites, namely, the mean coherent length, BET area and the inversion factor.

## 2. Materials and Methods

### 2.1. Preparation

All used chemicals, namely, FeCl3 · 6H2O, ZnCl_2_ and NaOH, were purchased from PENTA, s.r.o. (Prague, Czech Republic). All chemicals were of analytical grade and were used as purchased. A Panasonic microwave oven without inverter technology (nn–j155wb) with a maximum output power of 800 W, equipped with an additional reflux system and a shaft stirrer, was used as a source of microwaves. Two series, microwave-assisted (MW) and with the microwave field turned off (RT), of zinc ferrite nanoparticles were prepared by a coprecipitation. Stoichiometric amounts of ZnCl_2_ and FeCl3 · 6H2O were dissolved in deionized water in the presence of NaOH ($c=3\mathrm{mol}\;L^{-1}$). The total volume of the solution was 250 mL. No further control over the pH of the solution was necessary. After the set reaction time (see Table 1), the solution with the precipitate was diluted to 750 mL and cooled down in ice bath to stop any further evolution of the sample. Finally, the precipitate was filtered. The list of all samples and particular amounts of the used chemicals for each sample are provided in Table 1. The number given in the naming of the samples represents the reaction time in minutes, e.g., MW-10 means the microwave-assisted synthesis with the 10-minute reaction time.

### 2.2. X-ray Powder Diffraction

X-ray powder diffraction (XRD) was used for analyzing the crystal structure and phase composition of the studied samples. The measurements were conducted using a Bruker D8 ADVANCE powder diffractometer operating in the Bragg–Brentano parafocusing geometry. The diffractometer was equipped with a LYNXEYE position sensitive detector and with Co K${}_{\alpha }$ radiation source. A voltage of 35 kV and current of 40 mA were set for the X-ray tube. In addition, a 0.6 mm divergence slit and 2.5° axial Soller slits for the primary beam path and an Fe K${}_{\beta }$ filter and 2.5° axial Soller slits for the secondary beam path were applied for the measurements. The XRD patterns were measured in the 2θ range between 10° and 125° with the step of 0.02° (RT-60, RT-90 and MW-10 samples) and 0.05° (RT-30, MW-5, MW-20 and MW-30 samples).

The Rietveld refinement of the XRD patterns using the MAUD software [31] was applied to evaluate the lattice parameter and MCL of the samples. The isotropic size-strain model was used with the diffraction profiles described by a pseudo-Voigt function. A detailed description of the refinement and the fitted diffraction patterns can be found in Supplementary Materials.

### 2.3. Transmission ${}^{57}$Fe Mössbauer Spectroscopy

To distinguish different crystallographic sites and chemical states of ^57^Fe within the crystal structure in the samples, transmission Mössbauer spectroscopy measurements were conducted. A dual-channel Mössbauer spectrometer of our own design, “OLTWINS” [32], was used to collect the spectra. The spectrometer was equipped with ^57^Co radioactive source in Rh matrix with initial activity of 50 mCi (±10%). An α-Fe foil was used for a calibration of velocity axis. Low temperature (5 K) measurements in the external magnetic field (5 T) were performed with the Mössbauer spectrometer “OLTWINS” equipped with a custom-made cryostat (CRYOGENIC LIMITED, UK). MossWin 4.0 software was used for the spectra evaluation [33].

### 2.4. Electron Microscopy

VEGA3 LMU (TESCAN, Brno, Czech Republic) equipped with SDD XFlash 410-M (Bruker, Bremen, Germany) was used to measure the EDS spectra of the samples. The microscope uses Vega3 control software for imaging and QUANTAX Esprit 1.9. for elemental analysis. The primary beam energy was set to 30 keV for all the samples. The elemental analysis was applied to check the presence of both iron and zinc and to identify any additional impurities that appeared in the samples. The software analysis of EDS spectra tracked the signal of the elements at detection limit as well and the values of these elements are summed and presented as “balance”. Transmission electron microscopy (TEM) images were recorded using JEOL JEM 2100 (200 kV accelerating voltage). Prior to the measurements, the samples were dispersed in ethanol and added dropwise on a copper grid.

### 2.5. $N_{2}$ Adsorption Measurements

The BET area of the samples was determined by N_2_ adsorption measurements using Autosorb-iQ-C analyser (Quantachrome Anton Paar, Boynton Beach, FL, USA). All the adsorption isotherms were measured up to the saturation pressure by N_2_ at 77.4 K. The specific surface areas were calculated by a multipoint BET (Brunauer–Emmett–Teller) model over the relative pressure range selected by following the Rouquerol’s criteria. All the samples were outgassed at 25 °C for at least 12 h prior to the actual measurement.

### 2.6. Magnetometry

The samples were analyzed using a Quantum Design Physical Properties Measurement System (PPMS Dynacool system) with the vibrating sample magnetometer (VSM) option. The experimental data were corrected for the diamagnetism and the signal of the sample holder. The temperature dependence of the magnetisation was recorded in a sweep mode of 1 K min^−1^ in the zero-field-cooled (ZFC) and field-cooled (FC) measuring regimes. The hysteresis loops were recorded at temperatures of 5 K and 300 K in external magnetic fields ranging from −50 kOe to 50 kOe (in SI unit equal to −5 T to 5 T).

## 3. Results

### 3.1. X-ray Powder Diffraction

**Figure 1:**
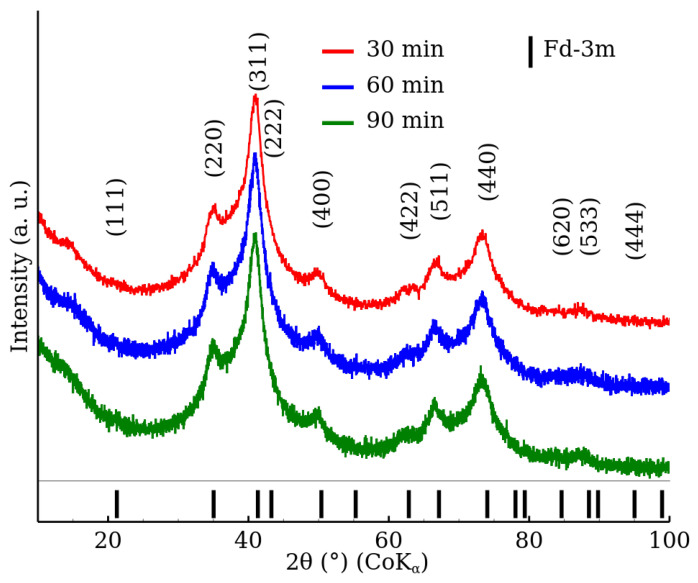
XRD patterns of zinc ferrite nanoparticles prepared with the microwave field turned off (samples RT-30, RT-60 and RT-90) in the 2θ range from 10° to 100°. The data were normalized with respect to the measurement time per step.

Figure 1 shows the X-ray diffraction patterns of the RT series samples, which were prepared at room temperature with no microwave field. A crystal structure exhibiting an Fd-3m space group, which is characteristic of a cubic spinel phase, was identified by the XRD analysis. In addition, the XRD patterns comprised significantly broadened diffraction peaks, which indicate a low crystallinity of the prepared samples. Table 2 shows the lattice parameter and MCL values obtained by the Rietveld refinement of the corresponding XRD patterns. The results show that the reaction time, within the studied time range, did not influence the crystalline properties of the samples. The obtained MCL values are in the nanometres range. Nonetheless, it should be noted that the exact numbers should be considered with care (especially for the RT series). The diffraction patterns exhibited a high degree of peak overlapping, and the refinement “misfits” were observed, which could notably influence the analysis results (see the Supplementary Materials).

**Figure 2:**
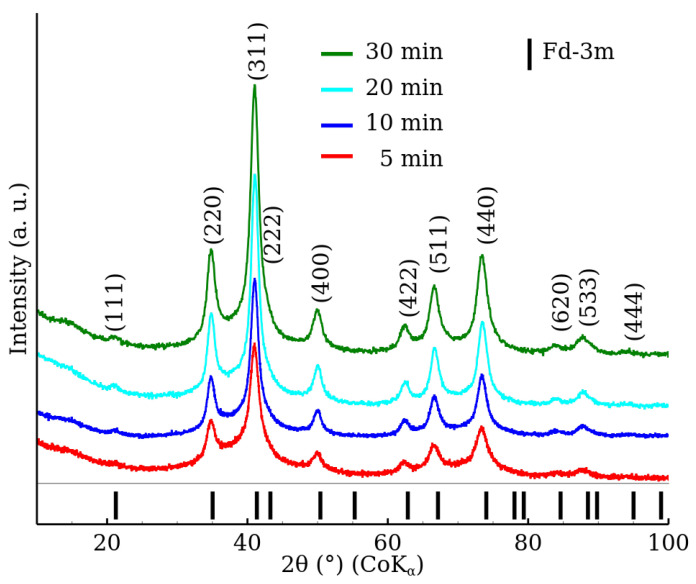
XRD patterns of zinc ferrite nanoparticles prepared by the microwave-enhanced approach (samples MW-5, MW-10, MW-20 and MW-30) in the 2θ range from 10° to 100°. The data were normalized with respect to the measurement time per step.

Similarly to the RT series patterns, the XRD patterns of the MW series samples show the presence of a crystal structure exhibiting the Fd-3m space group (Figure 2). However, in the case of the MW series, the diffraction patterns exhibited significantly narrower diffraction peaks. Subsequently, significantly higher MCL values were obtained (Table 2) in comparison to the RT series. The results show that a higher degree of crystallinity was achieved already after 5 min of microwave-assisted synthesis than after 90 min of reaction time without the microwave field. On the other hand, differences between the lattice parameter values are within the estimated uncertainties.

### 3.2. Transmission Electron Microscopy and Elemental Analysis

**Figure 3:**
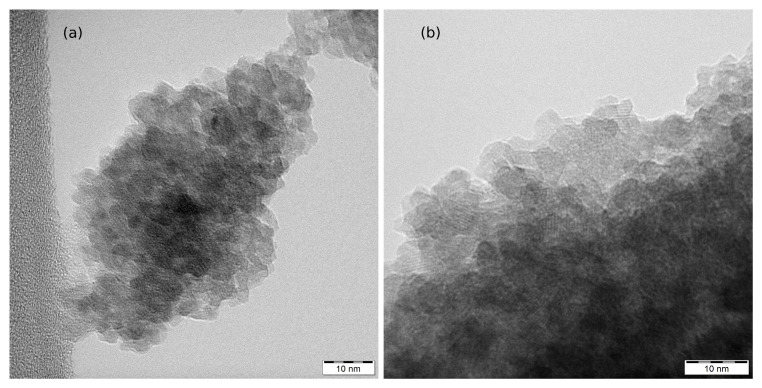
TEM images of measured nanoparticles and their aggregates: (**a**) RT-60 and (**b**) RT-90.

TEM images of RT-60 and RT-90 are shown in Figure 3. The images show highly aggregated spheroidal nanoparticles with sizes under 10nm. Nonetheless, the particle size distribution could not be precisely determined due to the high degree of overlapping among the individual nanoparticles.

**Figure 4:**
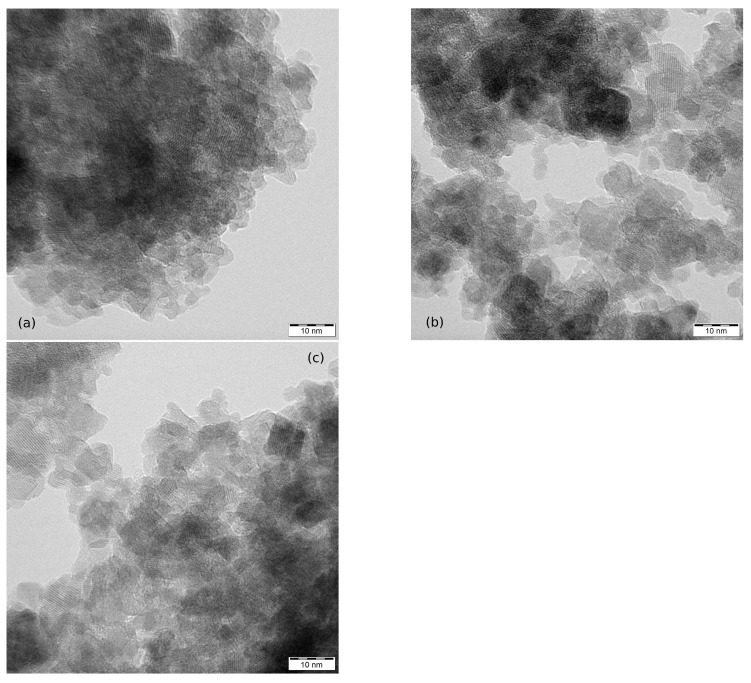
TEM images of measured nanoparticles and their aggregates: (**a**) MW-5, (**b**) MW-10 and (**c**) MW-30.

The images in Figure 4 show nanoparticles of MW-5, MW-10 and MW-30. Again the degree of aggregation did not allow the determination of the particle size distribution. The nanoparticles are under 10 nm in size with spheroidal shape. For additional TEM images see Supplementary Information.

**Figure 5:**
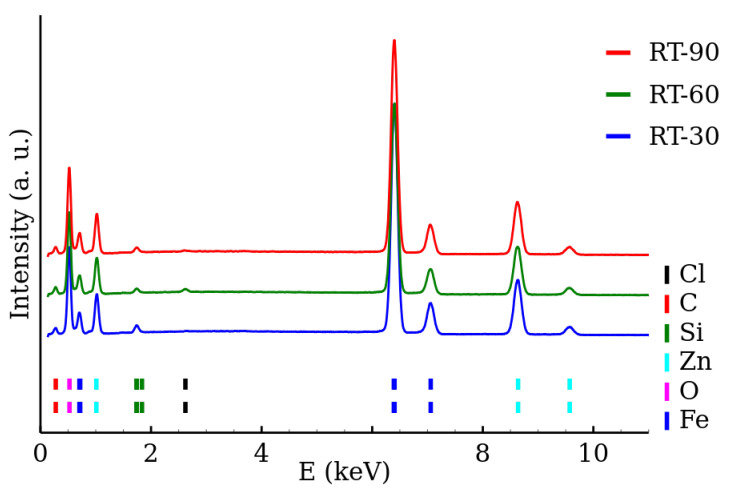
EDS spectra of room temperature synthesized zinc ferrite nanoparticles.

**Figure 6:**
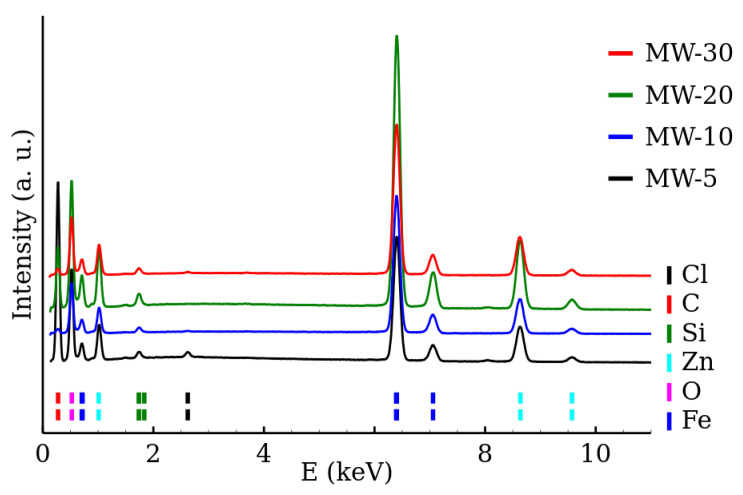
EDS spectra of zinc ferrite nanoparticles prepared by the microwave-enhanced synthesis.

The chemical composition of the samples was determined by the EDS. The EDS spectra of the RT series and MW series of samples are shown in Figure 5 and Figure 6, respectively. The presence of both zinc and iron was verified and the Fe/Zn stoichiometric ratio of 2.0±0.2 was found in all the samples (Table 3). The amount of other detected elements (other than Fe, Zn, O) did not exceed 2%. The EDS results are summarized in Table 3.

### 3.3. Specific Surface Area

**Figure 7:**
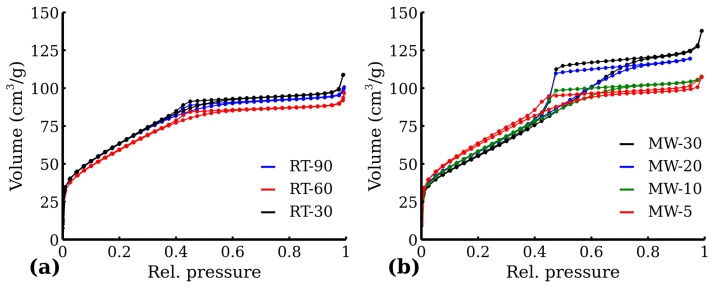
N_2_ adsorption isotherm of (**a**) RT samples and (**b**) MW samples.

The adsorption and desorption isotherms of all the samples were recorded and are shown in Figure 7. All RT samples in Figure 7a exhibited Type I curves supplemented with a small hysteresis curve. The BET results suggest a relatively high specific surface area over 200 m^2^ g^−1^ in the case of all the RT samples. Similarly to XRD, no significant differences were found among the samples of the RT series, although there was even 60-minute reaction time difference. On the other hand, a gradual change in MW series isotherms (Figure 7b) could be observed, from Type I (with a small hysteresis) to Type IV, signaling an evolution of the interparticle voids (and, indirectly, the particle growth). This correlates well with the results of XRD. Despite the said isotherm evolution, the BET area of the MW samples remained unaffected, as the BET isotherm region stayed unchanged. All the MW samples thus exhibited relatively high BET areas, comparable to those in the RT series. The BET values are shown in Table 4.

With the assumption of the uniform spherical and non-porous particles, a theoretical value of the specific surface area $S_{\mathrm{th}}$ could be determined using the equation [34]

(4) $$S_{\mathrm{th}}=\frac{6}{\rho D}\;,$$

where *D* is the mean diameter of the particles, and ρ is the density of the studied material. The density ρ can be calculated following the equation [35]:

(5) $$\rho =\frac{ZM}{Na^{3}}\;,$$

where *Z* is the number of molecules per unit cell (Z=8), *M* is the molar mass, *N* is the Avogadro constant and *a* is the lattice parameter characterizing a cubic lattice. For the *a* values determined from XRD the approximate value of ρ=5.3 g cm^−3^ was estimated. Using Equation (4) the estimation of the mean particle diameter *D* can be performed (assuming the uniform spherical and non-porous particles) area values from Table 4 gives *D* values of about 5–6 nm. This correlates well with both particle sizes observed in the TEM images and the MCL values.

### 3.4. Mössbauer Spectroscopy as a Tool for Determination of Inversion Factor

**Figure 8:**
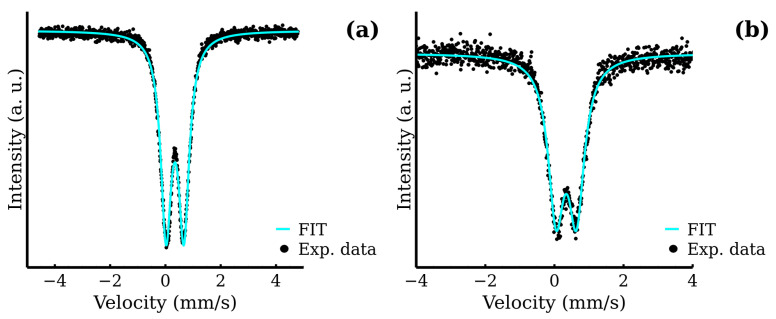
Room temperature Mössbauer spectra of MW samples: (**a**) MW-5 and (**b**) MW-30.

**Figure 9:**
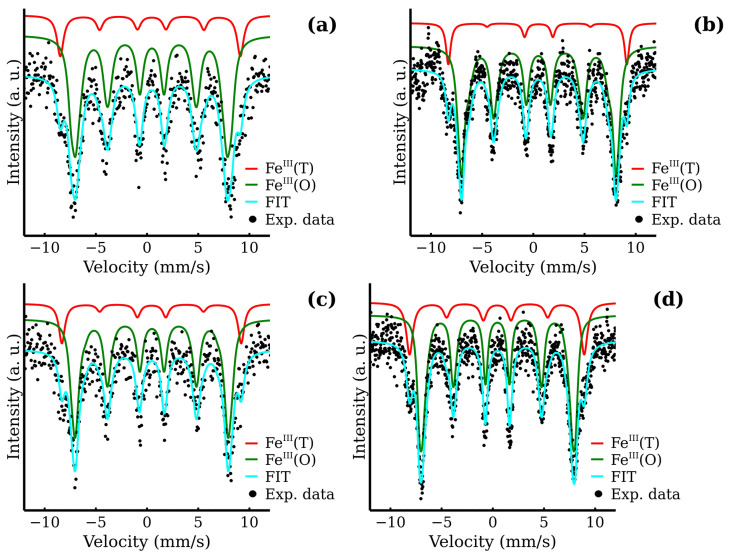
Low temperature Mössbauer spectra in the external magnetic field of MW samples: (**a**) MW-5, (**b**) MW-10, (**c**) MW-20 and (**d**) MW-30.

**Figure 10:**
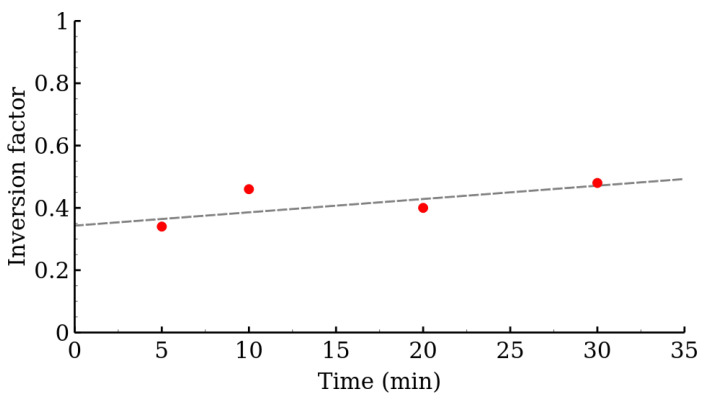
Calculated inversion factor of the MW series samples of zinc ferrite.

All room temperature Mössbauer spectra exhibited only one doublet. For the representative spectra, see Figure 8; for the rest of the spectra, see Supplementary Materials, Figures S8–S12. A small decrease in the quadrupole splitting with the increasing reaction time was observed in the MW series. This, in junction with other experimental results, may indicate the gradual ordering of the ferrite structure and increasing symmetry of Fe cations environment. The detailed list of hyperfine parameters is in Table 5. The low temperature Mössbauer spectroscopy in external magnetic field (5 T, 5 K) was employed to distinguish the Fe^III^ cations in the two crystallographic sites, i.e., tetrahedral and octahedral and to determine the inversion factor (see Figure 9). The in-field (5 K, 5 T) spectra consisted of two well-resolved sextets. These two components reflected the distribution of iron atoms between the tetrahedral and octahedral sites and confirmed a ferrimagnetic ordering of the samples. This distribution can be quantified through the relative spectral areas of the two sextets. The hyperfine parameters, as well as the relative spectral areas, are presented in Table 5. The relative areas of the two sextets can be easily used for the calculation of the inversion factor in accordance with Equations (1) and (3). The resulting values are shown in Figure 10. Displayed inversion factor values are in the interval 0.4±0.1 and are compared to a linear evolution. These values approach an ideal mixed spinel, where the zinc cations are equally distributed between the two crystallographic sites (inversion factor is 0.5).

### 3.5. Magnetometry

**Figure 11:**
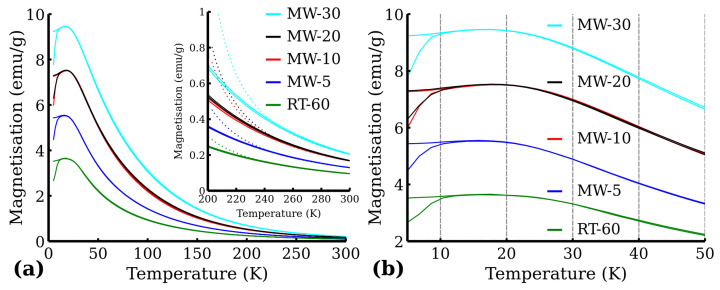
ZFC and FC (1000Oe) magnetization curves: (**a**) 5 K to 300 K range and (**b**) 5 K to 50 K range. Dotted lines correspond to the Curie–Weiss law curves.

**Figure 12:**
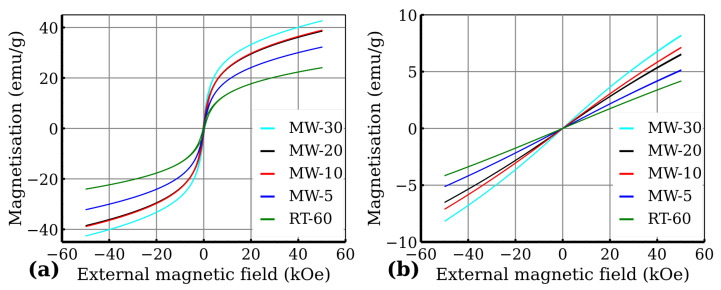
Hysteresis loops recorded at (**a**) 5 K and (**b**) 300 K.

The zero-field-cooled (ZFC) and field-cooled (FC) curves are presented in Figure 11. Generally, when the temperature decreases, the superspins of all the magnetic nanoparticles gradually freeze in the magnetically blocked regime. The temperature interval, where the transition to the magnetically blocked state occurs, is documented by the maximum at the ZFC magnetisation curve and represents the blocking temperature ($T_{B}$), i.e., the temperature at which the averaged-sized nanoparticles in the assembly are magnetically blocked ($T_{B,\mathrm{av}}$). The ZFC and FC magnetisation curves separate at the distinct temperature known as the temperature of irreversibility ($T_{\mathrm{irr}}$), which marks the onset of the blocking mechanism of superspins belonging to the largest nanoparticles in the system. Thus the difference between $T_{\mathrm{irr}}$ and $T_{B}$ can be described as a quantitative measure of the particle size distribution, if the magnetic anisotropy constant is known. The $T_{B}$ of the measured samples was around 15 K to 18 K for all measured samples with $T_{\mathrm{irr}}$ being very close to the maximum of the ZFC curve (Figure 11b). Moreover, below $T_{B}$, the FC magnetization values slightly decreased, which indicates that nanoparticles magnetically interact with each other very strongly. The FC–ZFC curve presented by Grasset et al. [36] has a similar shape, which they ascribed to aggregated nanoparticles, and they also observed that temperature of irreversibility is lower then blocking temperature due to ferrimagnetic behavior. To confirm the purity of all the samples from the magnetic point of view, as well as to confirm the superparamagnetic behavior of the samples, we also measured hysteresis loops at low temperature (5 K) and at room temperature (300 K)—see Figure 12. From the hysteresis loops, it is clearly evident that at 5 K, the samples show certain values of coercivity ($H_{C}$) and remanence ($M_{R}$), reflecting that the system is in the blocked state below the transition temperature. Room temperature magnetization measurements also show increases in susceptibility (Figure 12b). The values of remanence and coercivity at 300 K are significantly lower compared to those at 5 K (see Supplementary materials), which confirms the superparamagnetic state of the measured sample (the spins of all the magnetic nanoparticles fluctuate between the orientations of easy axis of magnetization) [37]. Additionally, the magnetic moment per ${Fe}^{\mathrm{III}}$ was determined using the Curie–Weiss law, which can be used for superparamagnetic nanoparticles in the case of high temperature in the form of:

(6) $$\chi =\frac{C}{T+\theta }\;,$$

where *C* is the Curie constant, *T* is the thermodynamic temperature, χ is the susceptibility and θ is the Weiss constant. The Curie constant thus serves to calculate the magnetic moment per ${Fe}^{\mathrm{III}}$$\mu_{eff}$ via the next equation:

(7) $$C=\mu_{0}N_{A}\frac{\mu_{\mathrm{eff}}^{2}}{3k_{B}}\;,$$

where $\mu_{0}$ represents the vacuum permeability and $k_{B}$ the Boltzmann constant [38]. The fitted Curie constant, Weiss constant and calculated values of magnetic moment per ${Fe}^{\mathrm{III}}$ are presented in Table 6. The magnetic moments per ${Fe}^{\mathrm{III}}$ are more than double the standard value of ${Fe}^{\mathrm{III}}$ magnetic moment ($\mu =5.92\mu_{B}$) except the RT-60 sample. The fitting curve is displayed for high temperature in Figure 11a). This further proves the superparamagnetic state of the prepared nanoparticles. The deviation in the MW-10 sample from the observed magnetization trend could be caused by the significant time difference between the measurement of MW-10 and the rest of the samples. The samples MW-5, MW-20, MW-30 and RT-60 might have slightly degraded over time.

## 4. Conclusions

The influence of microwave radiation on the synthesis and resulting properties of zinc ferrite nanoparticles was investigated. The ZnFe_2_O_4_ nanoparticles were prepared by a coprecipitation of Fe^3+^ and Zn^2+^, both with and without the assistance of microwave radiation. A direct comparison of the microwave-assisted samples with the samples that were prepared without the microwaves proves the considerable impact of the microwave field on the crystallinity of the prepared nanoparticles. A significant improvement in the crystallinity of the samples was observed already after 5–10 min of reaction time, whereas there was no observable difference after 90 min of reaction time at room temperature in the absence of the microwave field. The determination of the inversion factor revealed the mixed spinel structure in all of the samples. Additionally, the prepared zinc ferrite nanoparticles exhibited relatively high BET areas and superparamagnetic behavior. Microwave-assisted synthesis provides a fast, low-cost and green preparation method for zinc ferrite nanoparticles, which can be used in a number of ongoing applications, e.g., a catalysis or surface modifications. 

## Figures and Tables

**Table 1:** Samples prepared in microwave oven with reflux system with applied microwave field in $V(H_{2}O)=250\;mL$.

Sample	$m({\mathrm{FeCl}}_{3}\cdot 6H_{2}O)$	$m({\mathrm{ZnCl}}_{2})$	V(NaOH)	*t*	$P^{1}$
	(g)	(g)	(mL)	(min)	(W)
RT-30	0.541	0.136	3	30	0
RT-60	0.541	0.136	3	60	0
RT-90	0.541	0.136	3	90	0
MW-5	0.541	0.136	3	5	800
MW-10	0.541	0.136	3	10	800
MW-20	0.541	0.136	3	20	800
MW-30	0.541	0.136	3	30	800

^1^ Microwave output power.

**Table 2:** Quantification of XRD patterns of zinc ferrite samples.

Sample	Lattice Parameter ±0.02 **(Å)**	Mean Coherent Length **(Å)**
RT-30	8.49	28
RT-60	8.49	29
RT-90	8.49	29
MW-5	8.47	62
MW-10	8.47	78
MW-20	8.46	86
MW-30	8.47	77

**Table 3:** Quantification of EDS spectra of zinc ferrite samples in atomic concentration, where balance is the sum of elements, which are at detection limit.

	Fe	Zn	O	Si	Balance
Sample	±0.8	±0.5	±0.7	±0.3	±0.3
	(%)	(%)	(%)	(%)	(%)
RT-30	27.3	13.5	57.7	1.5	0.0
RT-60	25.8	12.5	60.5	1.0	0.2
RT-90	25.2	12.7	60.9	1.0	0.2
MW-5	19.7	10.1	68.3	1.0	0.9
MW-10	26.6	13.0	58.5	1.6	0.2
MW-20	25.1	12.9	60.1	1.7	0.2
MW-30	25.9	13.0	59.1	1.6	0.3

**Table 4:** Specific surface area of zinc ferrite samples.

Sample	BET Area	Type of Isotherm
	±7%	IUPAC
	(m${}^{2}$ g${}^{-1}$)	Classification
RT-30	225	I
RT-60	220	I
RT-90	234	I
MW-5	231	I
MW-10	217	I/IV
MW-20	214	IV
MW-30	207	IV

**Table 5:** Components and parameters of room temperature Mössbauer spectra of prepared samples.

Sample	δ	$\Delta E_{Q}$	$B_{\mathrm{eff}}$	FWHM	RA	Identification
	±0.01	±0.01	±0.3	±0.01	±2	
	(mm s${}^{-1}$)	(mm s${}^{-1}$)	(mm s${}^{-1}$)	(mm s${}^{-1}$)	(%)	
RT-30	0.34	0.65	—	0.45	100	Fe^III^
RT-60	0.35	0.66	—	0.46	100	Fe^III^
RT-90	0.35	0.67	—	0.48	100	Fe^III^
MW-5	0.35	0.65	—	0.47	100	Fe^III^
MW-5	0.39	−0.16	52.6	0.91	33	Fe^III^(T)
(5 K, 5 T)	0.45	−0.10	46.0	0.62	67	Fe^III^(O)
MW-10	0.35	0.64	—	0.47	100	Fe^III^
MW-10	0.40	−0.24	53.0	0.84	27	Fe^III^(T)
(5 K, 5 T)	0.46	−0.03	46.3	0.56	73	Fe^III^(O)
MW-20	0.34	0.62	—	0.47	100	Fe^III^
MW-20	0.41	−0.12	52.8	0.76	30	Fe^III^(T)
(5 K, 5 T)	0.46	−0.04	46.3	0.52	70	Fe^III^(O)
MW-30	0.34	0.59	—	0.55	100	Fe^III^
MW-30	0.41	−0.03	53.0	0.77	26	Fe^III^(T)
(5 K, 5 T)	0.46	0.00	45.9	0.51	74	Fe^III^(O)

*δ* is an isomer shift, Δ*E_Q_* is a quadruple splitting energy, *B*_eff_ effective magnetic field and RA is relative area.

**Table 6:** Fitted parameters of Curie–Weiss law.

Sample	Curie Constant	Weiss Constant	$\mu_{\mathrm{Fe}}$
	(emu K Oe g${}^{-1}$)	(K)	($\mu_{B}$)
RT-60	$1.39\times 10^{-2}$	$-1.54\times 10^{2}$	10.5
MW-5	$1.77\times 10^{-2}$	$-1.64\times 10^{2}$	11.9
MW-10	$2.14\times 10^{-2}$	$-1.73\times 10^{2}$	13.1
MW-20	$2.08\times 10^{-2}$	$-1.77\times 10^{2}$	12.9
MW-30	$2.41\times 10^{-2}$	$-1.83\times 10^{2}$	13.9

## Data Availability

The data are available on request from the corresponding author.

## Supplementary Materials

The following supporting information can be downloaded at: https://www.mdpi.com/article/10.3390/nano12172987/s1, Figure S1: XRD pattern and Rietveld fit of sample RT-30; Figure S2: XRD pattern and Rietveld fit of sample RT-60; Figure S3: XRD pattern and Rietveld fit of sample RT-90; Figure S4: XRD pattern and Rietveld fit of sample MW-5; Figure S5: XRD pattern and Rietveld fit of sample MW-10; Figure S6: XRD pattern and Rietveld fit of sample MW-20; Figure S7: XRD pattern and Rietveld fit of sample MW-30; Figure S8: Room temperature Mössbauer spectra RT-30; Figure S9: Room temperature Mössbauer spectra RT-60; Figure S10: Room temperature Mössbauer spectra RT-90; Figure S11: Room temperature Mössbauer spectra MW-10; Figure S12: Room temperature Mössbauer spectra MW-20; Figure S13: TEM images of sample RT-60; Figure S14: TEM images of sample RT-90; Figure S15: TEM images of sample MW-5; Figure S16: TEM images of sample MW-10; Figure S17: TEM images of sample MW-30; Figure S18: Hysteresis loops of sample RT-60; Figure S19: Hysteresis loops of sample MW-5; Figure S20: Hysteresis loops of sample MW-10; Figure S21: Hysteresis loops of sample MW-20 and Figure S22: Hysteresis loops of sample MW-30.
