# Supplementary material for: Microwave-Enhanced Crystalline Properties of Zinc Ferrite Nanoparticles
Source: Nanomaterials (Basel). 2022 Aug 29;12(17):2987. doi: 10.3390/nano12172987 (PMC9457733; doi:10.3390/nano12172987)
Supplement: Supplementary file 1 [file nanomaterials-12-02987-s001.zip › nanomaterials-1814152-suppl.pdf]

Supplementary Materials: Microwave-enhanced crystalline properties of zinc ferrite nanoparticles

Martin Ochmann [1,‡], Vlastimil Vrba [1,‡], Josef Kopp [1,‡], Tomáš Ingr [1], Ondřej Malina [2] and Libor Machala [1,‡*]

[1] Department of Experimental Physics, Faculty of Science, Palacký University Olomouc, 17. listopadu 1192/12, 779 00 Olomouc, Czech Republic
[2] Regional Centre of Advanced Technologies and Materials, Czech Advanced Technology and Research Institute (CATRIN), Palacký University Olomouc, Šlechtitelů 27, 779 00 Olomouc, Czech Republic
* Correspondence: libor.machala@upol.cz
‡ These authors contributed equally to this work.

Rietveld refinement of the X-ray diffraction patterns

The Rietveld refinement was applied for the XRD data corresponding to each sample from the RT series and the MW series. The data were fitted in the range from 15° to 125°. To model the background a polynomial function of degree 4 was used together with a background peak (a Gaussian function helping to describe the background in the lowest angle region). A standard Delft line broadening model was used in the MAUD software.

The refined material parameters were the lattice parameter, MCL (apparent crystallite size), and the isotropic temperature factor (set as common to all atoms). The isotropic strain parameter was set to zero, the relative coordinates of oxygen atoms were set to $x_O = y_O = z_O = 0.25$, and the cation distribution with $\mathrm{Zn : Fe : O} = 1 : 2 : 4$ and the inversion degree $x = 0.75$ was considered.

It should be noted that the exact values of the mentioned fixed parameters should be taken with caution due to the model imperfections. For example, the inversion factor $x = 0.75$ is higher than the values obtained from the Mössbauer spectroscopy measurements (which resulted in worse fit quality). This could, however, be an effect of minimizing the "misfits" (deviations between the experimental data and theoretical curves), which could actually be caused by other phenomena. The fit quality parameters R_{wp} and GoF (goodness of fit) are listed for each refined dataset. The observed misfits (see the residua) are similar for all samples from both RT and MW series. The XRD patterns exhibited a significant peak overlapping, which could not be ideally described using the standard profile and background functions within the considered model.

Regarding the lattice parameter, the refinement imperfections were reflected by the high uncertainties, which were estimated by comparing results from several different models. The MCL was refined (i) to inspect the nano-scale of the zinc ferrite particles, and (ii) to study the relative changes in the MCL values as the result of the applied microwave radiation. These points are clearly shown by the results. Nevertheless, the resulting values could be affected by the fitting imperfections and an estimation of the MCL uncertainties is very complicating. Possible causes of the misfits might include particle size distributions or partially amorphous regimes in the samples affecting the XRD patterns background.

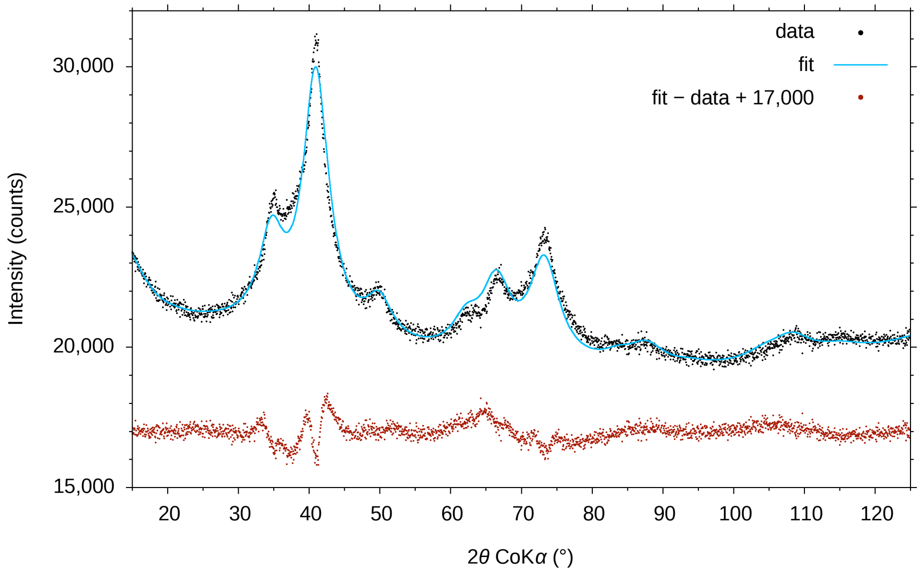

Figure S1. XRD pattern and Rietveld fit of sample RT-30 ($R_{wp} = 1.38\,\%$, GoF = 2.10).

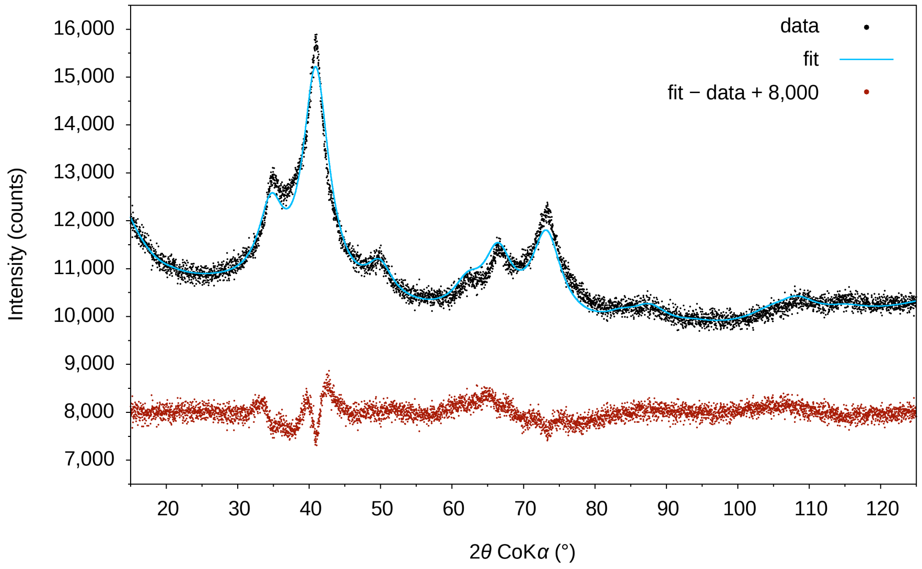

Figure S2. XRD pattern and Rietveld fit of sample RT-60 ($R_{wp} = 1.55\,\%$, GoF = 1.62).

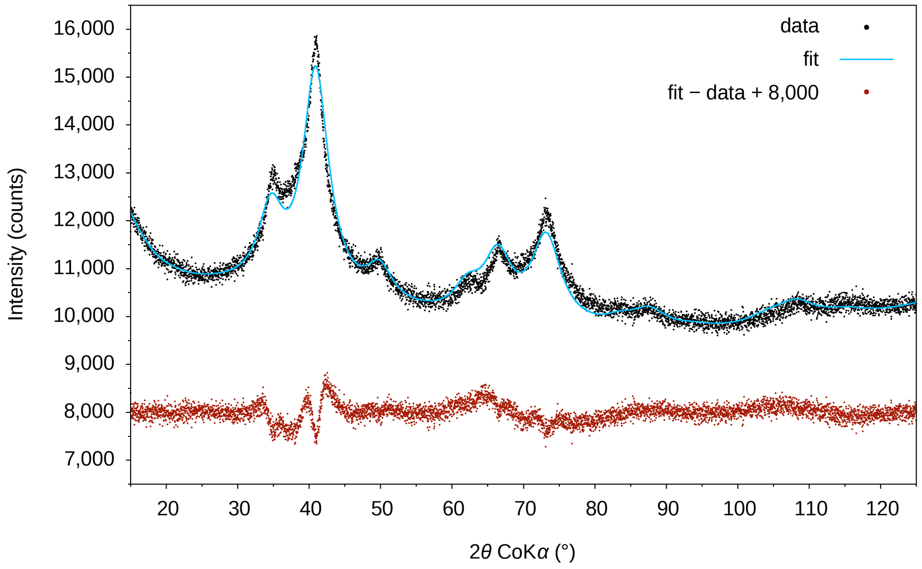

Figure S3. XRD pattern and Rietveld fit of sample RT-90 (R_{wp} = 1.58 %, GoF = 1.65).

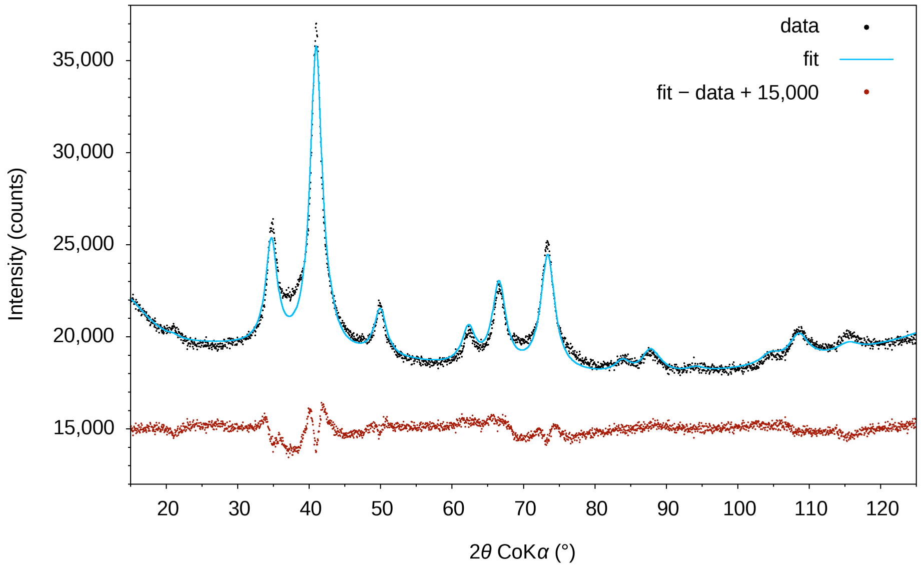

Figure S4. XRD pattern and Rietveld fit of sample MW-5 (R_{wp} = 1.64 %, GoF = 2.33).

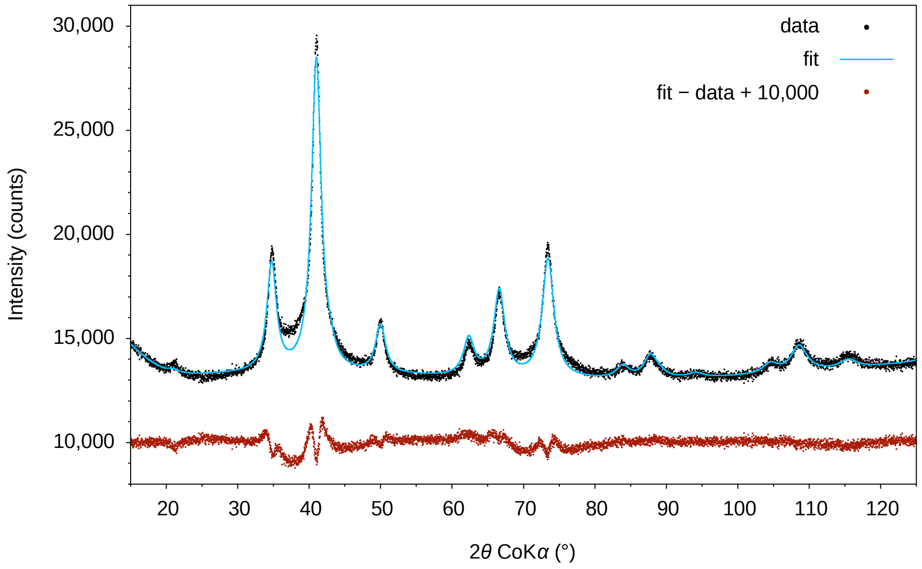

Figure S5. XRD pattern and Rietveld fit of sample MW-10 (R_{wp} = 1.79 %, GoF = 2.14).

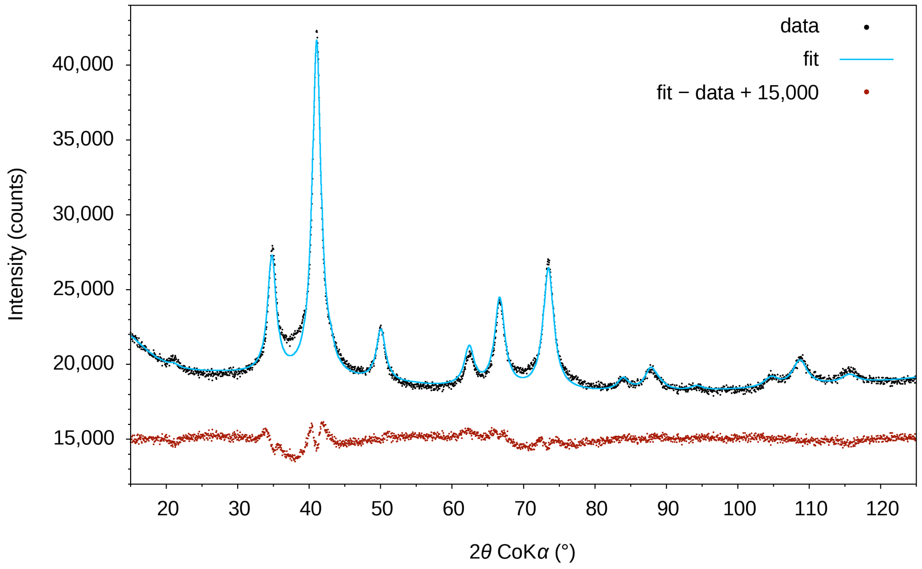

Figure S6. XRD pattern and Rietveld fit of sample MW-20 (R_{wp} = 1.55 %, GoF = 2.20).

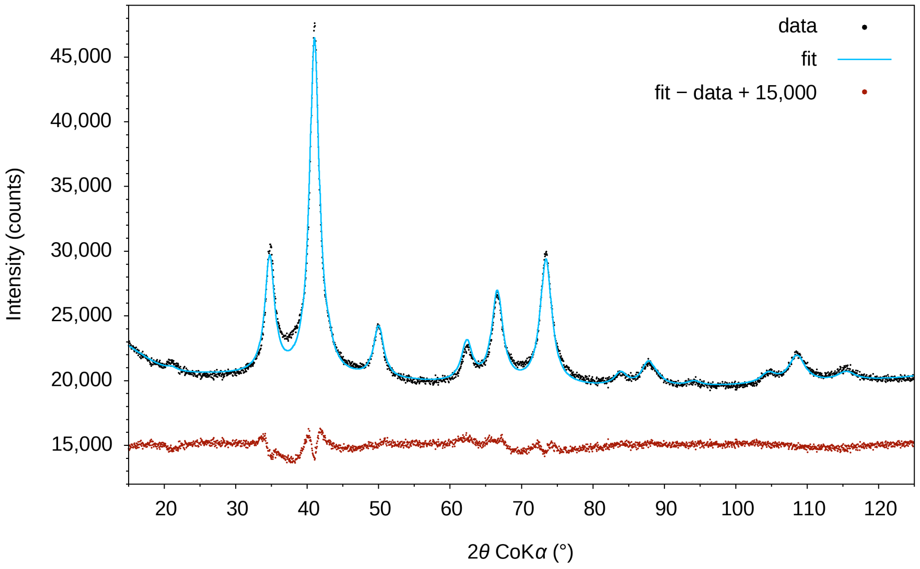

Figure S7. XRD pattern and Rietveld fit of sample MW-30 ($R_{wp} = 1.43\,\%$, GoF = 2.10).

Room temperature Mössbauer spectra

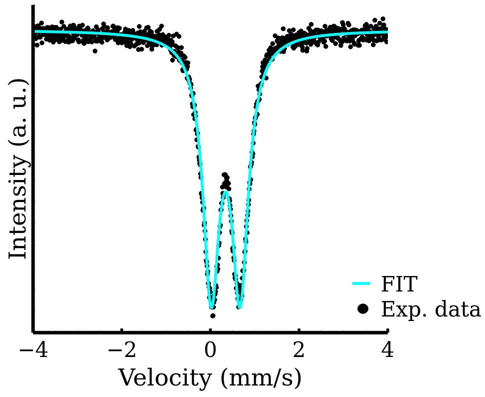

Figure S8. Room temperature Mössbauer spectra RT-30.

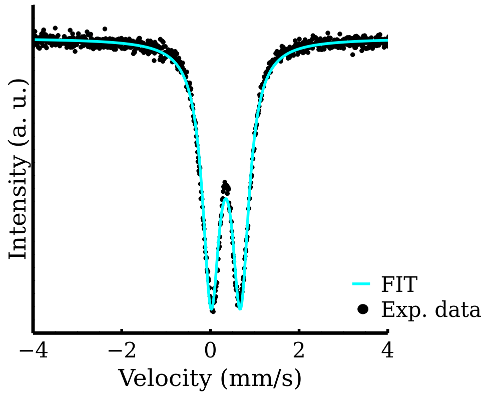

Figure S9. Room temperature Mössbauer spectra RT-60.

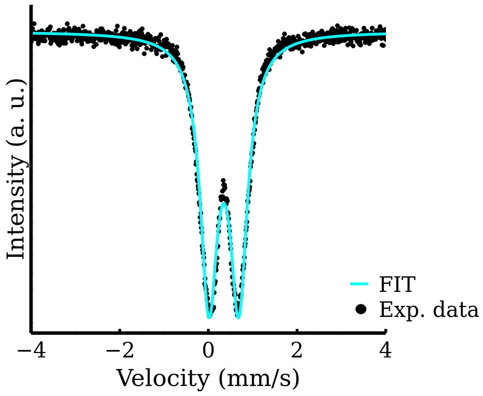

Figure S10. Room temperature Mössbauer spectra RT-90.

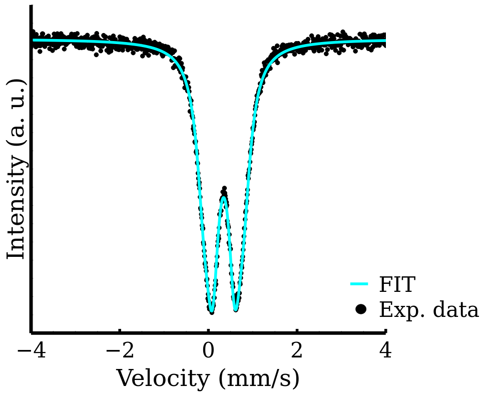

Figure S11. Room temperature Mössbauer spectra MW-10.

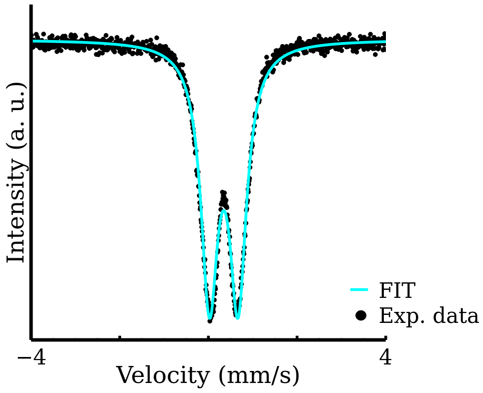

Figure S12. Room temperature Mössbauer spectra MW-20.

TEM images of measured samples

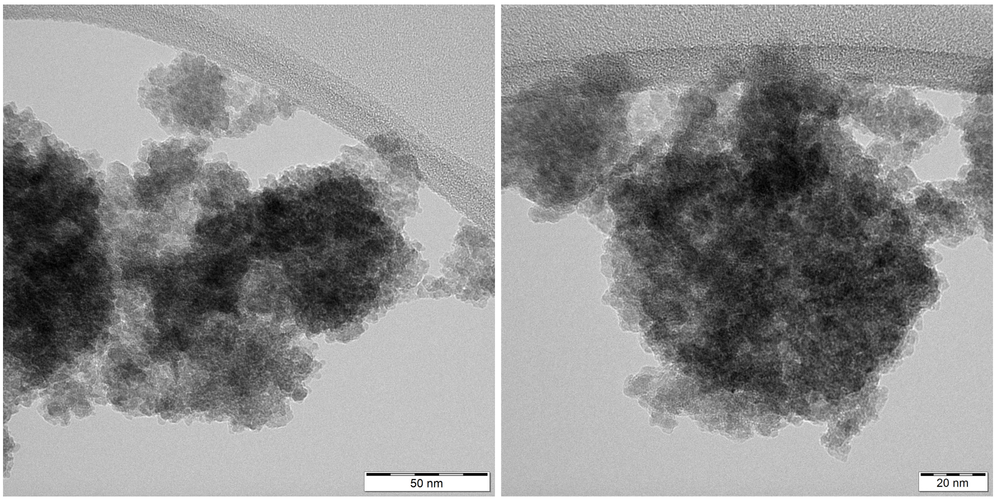

Figure S13. TEM images of sample RT-60

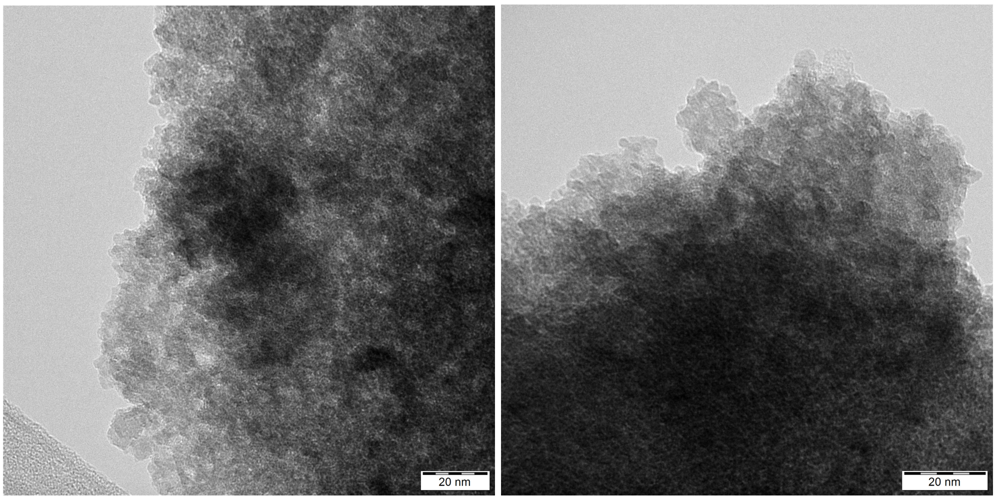

Figure S14. TEM images of sample RT-90

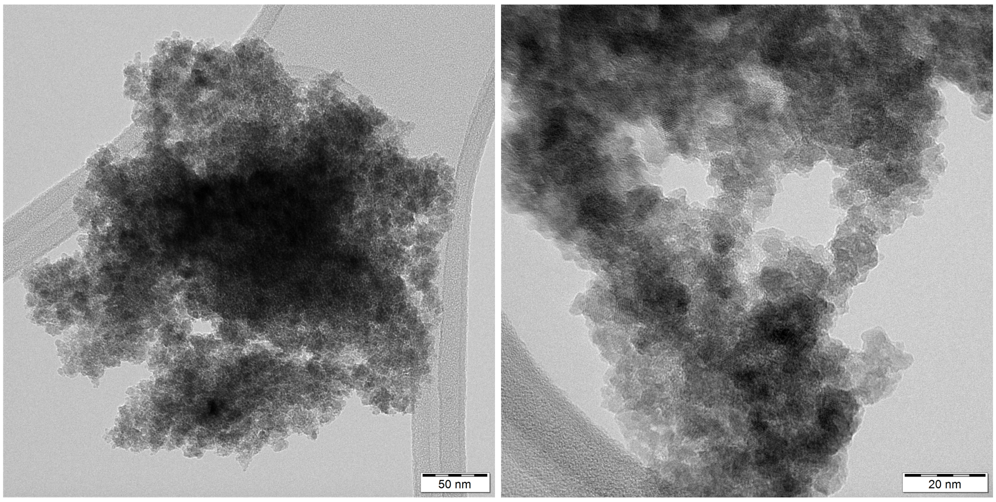

Figure S15. TEM images of sample MW-5

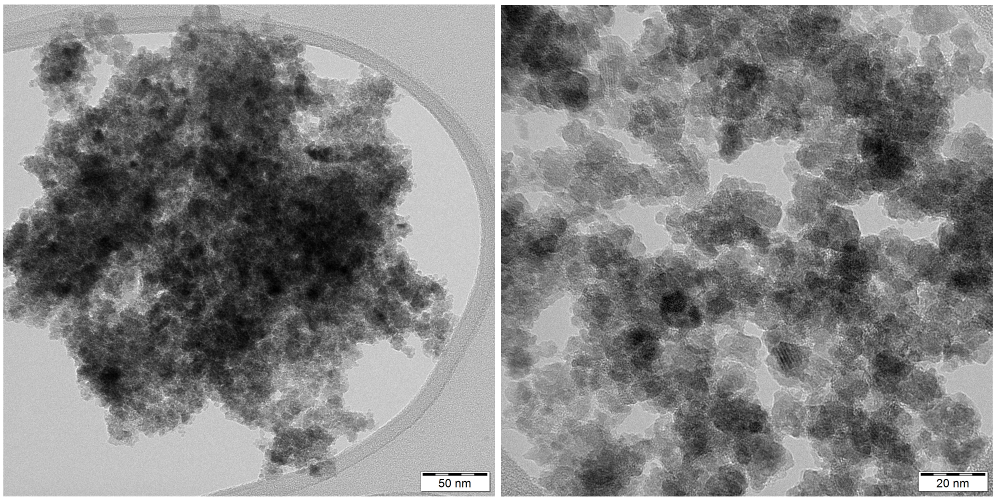

Figure S16. TEM images of sample MW-10

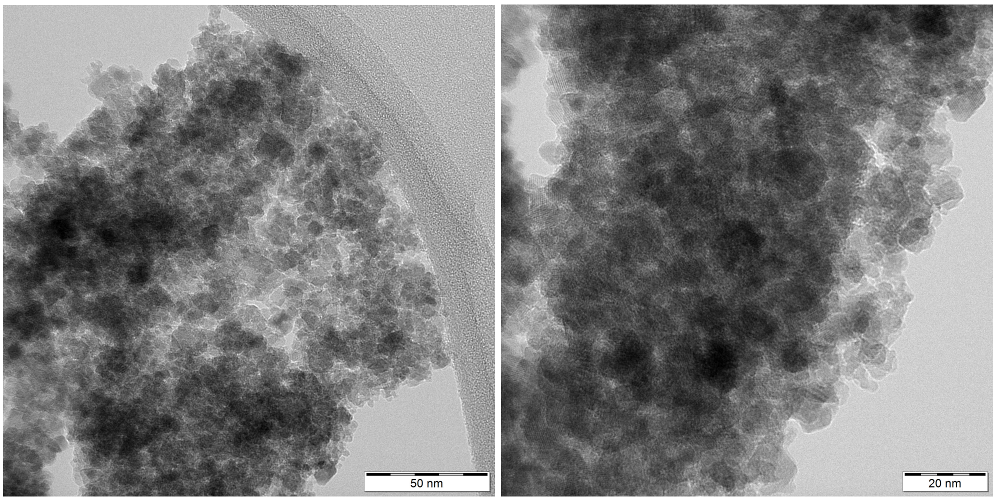

Figure S17. TEM images of sample MW-30

Hysteresis loops at 5 K and 300 K

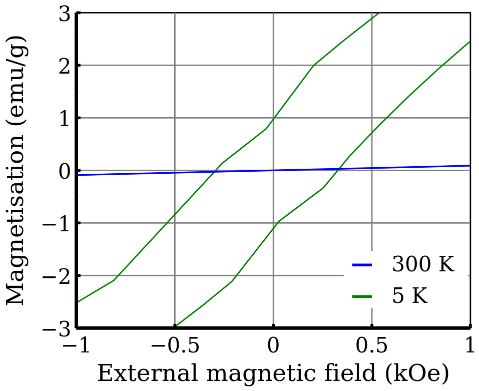

Figure S18. Hysteresis loops of sample RT-60.

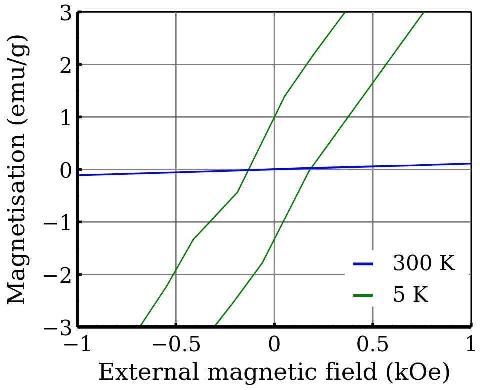

Figure S19. Hysteresis loops of sample MW-5.

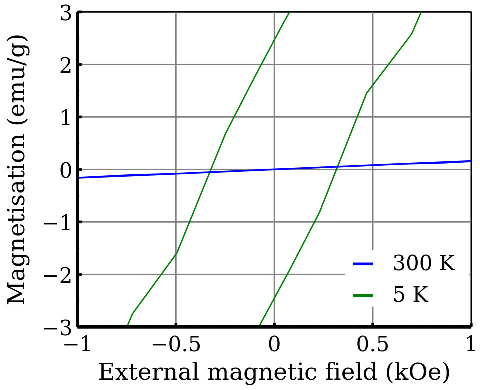

Figure S20. Hysteresis loops of sample MW-10.

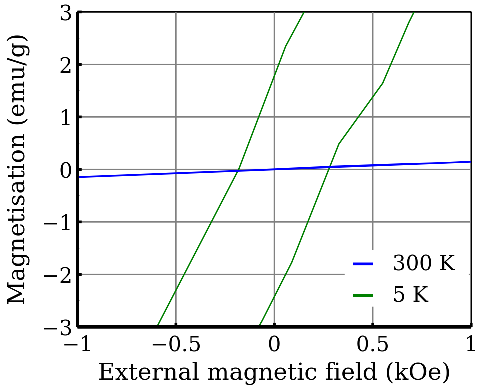

Figure S21. Hysteresis loops of sample MW-20.

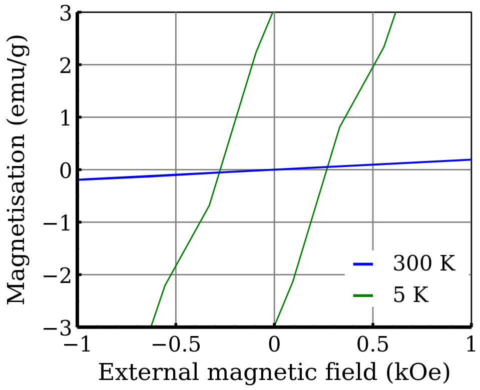

Figure S22. Hysteresis loops of sample MW-30.
